# The impact of the COVID-19 pandemic on incidence and clinical presentation of thrombotic microangiopathies: data from a laboratory centralizing ADAMTS-13 testing in Quebec

**DOI:** 10.1186/s13023-025-03960-5

**Published:** 2025-09-24

**Authors:** Clémence Merlen, Sandrine Thouzeau-Benghezal, Emmanuelle Pépin, Samuel Guay, Anne-Laure Lapeyraque, Alexandra Cambier, Georges-Etienne Rivard, Stéphan Troyanov, Arnaud Bonnefoy

**Affiliations:** 1 Division of Hematology-Oncology, CHU Sainte-Justine, Université de Montréal, 3175, chemin de la Côte-Ste-Catherine, Montreal, Quebec H3T 1C5 Canada; 2 Department of Clinical Laboratory Medicine, OPTILAB Montréal-CHU Sainte-Justine, Montreal, QC Canada; 3 Department of Psychology, Université de Montréal, Montreal, QC Canada; 4 Division of Nephrology, CHU Sainte-Justine, Université de Montréal, Montreal, QC Canada; 5 Division of Nephrology, Hôpital du Sacré-Coeur, Université de Montréal, Montreal, QC Canada

**Keywords:** Thrombotic microangiopathy, Thrombotic thrombocytopenic purpura, ADAMTS-13, Incidence, Epidemiology, COVID-19

## Abstract

**Background:**

Coronavirus disease 2019 (COVID-19) primarily induces respiratory symptoms. However, some patients develop thrombotic microangiopathies (TMA) during their infection. This study aimed to compare the incidence and clinical presentation of patients referred for TMA suspicion in Quebec (Canada) before and during the COVID-19 pandemic, utilizing data from a laboratory centralizing ADAMTS-13 testing.

**Results:**

During the pre-pandemic period (2017–2019), 500 patients were referred for TMA suspicion, of whom 423 had no prior TMA episodes. Among these, 50 patients exhibited ADAMTS-13 activity ≤ 10%, confirming a diagnosis of thrombotic thrombocytopenic purpura (TTP). In the pandemic period (2020–2022), 683 patients were referred for TMA suspicion, with 600 experiencing their first TMA episode. TTP was identified in 53 patients. In our cohort, the mean incidence of TTP cases remained steady between the pre-pandemic and the pandemic period (approximately 2 cases per million persons per year). Females with TTP were younger during the pandemic compared to the pre-pandemic period (median age 41.5 vs. 51.5 years; *p* = 0.032). Overall, the clinical presentation of TTP and suspected TMA other than TTP patients remained comparable between the two periods. The mean incidence of suspected TMA other than TTP cases increased in males during the pandemic compared to the previous period (20.3 vs. 12.7 per million persons per year; *p* = 0.023), particularly in men aged 50–59 years (28.7 vs. 11.9 per million persons per year; *p* = 0.05). A weak cross-correlation between new COVID-19-related hospitalizations and new cases of suspected TMA other than TTP was observed, peaking at a lag of 1 week (*r* = 0.258). COVID-19-associated TMA was suspected in 17 patients, with 3 cases confirmed as TTP based on ADAMTS-13 activity results. Patients with suspected TMA other than TTP associated with COVID-19 were predominantly male (86%) with a median age of 53 years (IQR: 43.2–69.2).

**Conclusion:**

The incidence of TTP did not change during COVID-19 pandemic. An increase referral of other suspected TMA was observed in our cohort, especially in males.

**Supplementary Information:**

The online version contains supplementary material available at 10.1186/s13023-025-03960-5.

## Background

Thrombotic microangiopathies (TMAs) are a group of rare hematologic disorders sharing clinical manifestations such as peripheral thrombocytopenia, microangiopathic hemolytic anemia (MAHA) and end-organ ischemia (e.g. brain, heart, kidneys) due to capillaries and small arteries thromboses [1–3]. The main subtypes of TMA are thrombotic thrombocytopenic purpura (TTP), typical haemolytic uremic syndrome (HUS) and complement-mediated TMA (CM-TMA), each distinguished by different underlying pathophysiological mechanisms [3, 4]. TTP is caused by a severe deficiency of ADAMTS-13 (a disintegrin and metalloproteinase with a thrombospondin type 1 motif, member 13), a von Willebrand factor (VWF) cleaving protease. TTP is suspected when ADAMTS-13 activity is ≤ 10% and is typically acquired due to auto-antibodies against ADAMTS-13 (approximately 95% of cases) but can also be congenital (Upshaw-Schulman syndrome) due to mutations on the corresponding gene affecting ADAMTS-13 function [5, 6]. HUS, mainly caused by Shiga toxin-producing *Escherichia coli* (STEC-HUS), is usually associated with bloody diarrhea, severe renal impairment and normal or slightly reduced ADAMTS-13 activity [7, 8]. CM-TMA involves genetic mutations or autoantibodies against regulating factors of the complement alternative pathway, leading to increased complement activity [1, 7]. TMA can occur spontaneously or secondary to underlying conditions such as pregnancy, autoimmune disease, malignancy, transplantation, infection or use of specific treatments [2, 9, 10]. The management of TMA may vary based on the TMA form and is guided by ADAMTS-13 activity assays [11–13]. Secondary TMA forms may also require treatment of the underlying cause [13].

The severe acute respiratory syndrome coronavirus 2 (SARS-CoV-2), the causative agent of coronavirus disease 2019 (COVID-19), is primarily associated with respiratory symptoms but can also lead to coagulopathy and diverse systemic complications affecting organs such as the lungs, kidneys, and heart [14–16]. Hemostatic alterations observed in COVID-19 include increased levels of coagulation factor VIII, elevated D-dimer, fibrinogen, and VWF [17]. Case reports have documented COVID-19-associated TMAs, including both TTP and CM-TMA forms [18–23]. It has been suggested that SARS-COV-2 may induce TMA through several mechanisms including direct or complement-mediated endothelial injury and platelet activation [19, 20]. Additionally, a substantial proportion of patients with COVID-19 developed autoantibodies to ADAMTS-13 during the course of the disease [24]. However, some studies indicated that classic TMA features, such as thrombocytopenia, hemolytic anemia or schistocytes were generally absent in COVID-19 patients, even among those exhibiting signs of coagulopathy. While mild to moderate reductions in ADAMTS-13 activity have been observed, possibly associated with markedly increased VWF levels, severe ADAMTS-13 deficiency appears to be rare in COVID-19 [25, 26]. In addition to SARS-CoV-2 infection, several case reports suggested that COVID-19 vaccination may also trigger TMA [27]. However, larger studies have not found a significant increase in the risk of immune TTP following vaccination [28, 29], nor have they identified COVID-19 vaccination as a strong trigger for acute episodes in patients with hereditary TTP [30].

The annual incidence of TTP is estimated to be between 1,5 and 6 cases per million population [31–34], while the incidence of CM-TMA ranges from 0.2 to 1,9 cases per million population [35]. In Canada, the annual incidence of TTP varies from 1 to 3.2 cases per million population [36–39]. This study aimed to assess the impact of the COVID-19 pandemic on the incidence of TMAs in the Canadian province of Quebec (population ~ 8.6 million). Using data from a centralized laboratory conducting ADAMTS-13 testing across the province, we estimated TMA incidence and compared the initial clinical presentation of TTP patients and those referred for TMA suspicion before and during the pandemic. The latter group, referred in our study as “suspected non-TTP TMA” may include various conditions such as STEC-HUS, CM-TMA, and other disorders with TMA-like features for which physicians considered ADAMTS-13 activity testing clinically relevant. Although this group is not well-defined, we included it in our study to gain a more comprehensive understanding of referral patterns during the two periods studied.

## Material and methods

### Selection of patients

The study was performed as described previously [37]. Briefly, plasma samples from all patients with a suspicion of TMA in the province of Quebec are tested for ADAMTS-13 activity at CHU Sainte-Justine (CHUSJ) hemostasis laboratory since 2013, when it has been designated as the only supplier of this test for this province by the *Institut National d’Excellence en Santé et Service* sociaux (INESSS). The incidence of TMAs was compared between two intervals of 3 years each. Data between January 1, 2017, and December 31, 2019, was considered the pre-pandemic period. Data between January 1, 2020, and December 31, 2022, was considered the pandemic period. The Research Ethics Committee of CHUSJ approved this study (#2019–2184).

### Data collection

Demographic data and laboratory results were extracted from the laboratory information system (SoftLab v4.0.8.3.7; SCC soft computer, Florida). Clinical data were collected from the medical questionnaire (F-726 form), which physicians are required to complete when requesting ADAMTS-13 activity testing as described previously [37]. This form includes information on associated pathologies, the clinical context indicating an acute stage, biological parameters, treatments and the date of any previous TMA episode, if applicable. Because specimens were sent to CHUSJ to confirm or exclude TTP and for the follow-up of TTP patients by measuring ADAMTS-13 activity, information collected regarding HUS and CM-TMA diagnoses was only partial, which limited the interpretability of data related to TMAs other than TTP. Data on the number of new COVID-19-related hospitalizations (incident hospitalizations) were obtained from the *Institut National de Santé Publique du Québec (INSPQ)* [40]. Incident hospitalizations included all hospital stays in which a diagnosis of COVID-19 was established either at the time of admission or during hospitalization. This category encompasses patients hospitalized primarily for COVID-19 and patients diagnosed with COVID-19 who were admitted for another reason.

### Classification of patients according to ADAMTS-13 activity level

Patients referred for ADAMTS-13 testing due to clinical suspicion of TMA by their respective physicians were categorized into three groups based on their functional ADAMTS-13 activity at presentation before the initiation of plasma exchange treatment as follows: (1) the supported TTP group: patients with ADAMTS-13 activity ≤ 10% with or without confirmation of an anti-ADAMTS-13 antibody; (2) the non-TTP TMA group: patients with suspected TMA other than TTP, exhibiting ADAMTS-13 activity > 10% at presentation and at intervals away from plasmapheresis. Due to the lack of confirmatory diagnoses for HUS and CM-TMA, the non-TTP TMA group may also encompass patients with TMA-like presentations; (3) the non-categorizable group: patients with a suspected TMA and ADAMTS-13 activity > 10% for whom the timing between plasmapheresis and plasma sample collection was unknown. This latter group can include both TTP and suspected non-TTP TMA patients.

### Data analysis

Incidence was calculated by dividing the number of newly identified cases by the mid-year estimated population of Quebec of the related years. Population estimates were obtained from Statistics Canada [41]. Patients with a documented previous TMA episode occurring either within or outside the study period were excluded from the incidence calculation. Each new case was categorized by age group, gender, and year of occurrence to calculate age-specific and sex-specific incidence rates of the related years. The incidence for the pre-pandemic and COVID-19 periods was calculated as the mean of the annual incidence within each respective 3-year period.

The normalized test rate was determined by dividing the number of tests performed by the total number of patients in the corresponding year. This rate was calculated exclusively for TTP patients, as ADAMTS-13 activity testing is utilized at diagnosis, during follow-up, and at recurrences in this group. In contrast, for patients with TMA other than TTP, ADAMTS-13 activity testing is usually performed only at presentation to rule out TTP.

### ADAMTS-13 activity measurement

ADAMTS-13 activity was assessed by measuring the cleavage of an ADAMTS-13-specifc fluorescent FRETS-VWF73 substrate (Peptide Institute, Osaka, Japan) [42] on a Synergy 4 microplate reader (BioTek, Winooski, VT).

### Statistical methods

Statistical analyses were performed using IBM SPSS statistics (version 28.0.0.0). Baseline characteristics are presented as mean and standard deviation (SD) or median and interquartile range (IQR) where specified. Qualitative data are expressed as percentages. Continuous variables between the pre-COVID-19 and pandemic periods were compared using the Student’s independent t-test or the Mann-Whitney U test. Cliff’s Delta (δ) was used to estimate effect size. Values of 0.147, 0.33 and 0.474 were interpreted as indicating small, medium, and large effect sizes, respectively. Subgroup analyses by age category were conducted using one-way ANOVA. When statistical significance was observed, Bonferroni-adjusted p values were reported. Categorical variables were compared using the χ2 test or Fisher’s exact test. COVID-19-related hospitalizations and new investigations of TMAs during the pandemic period were cross-correlated using the cross-correlation function (CCF). Statistical significance was defined as a p-value of 0.05 or lower. All tests were two-sided.

## Results

### Study population

**Fig. 1:**
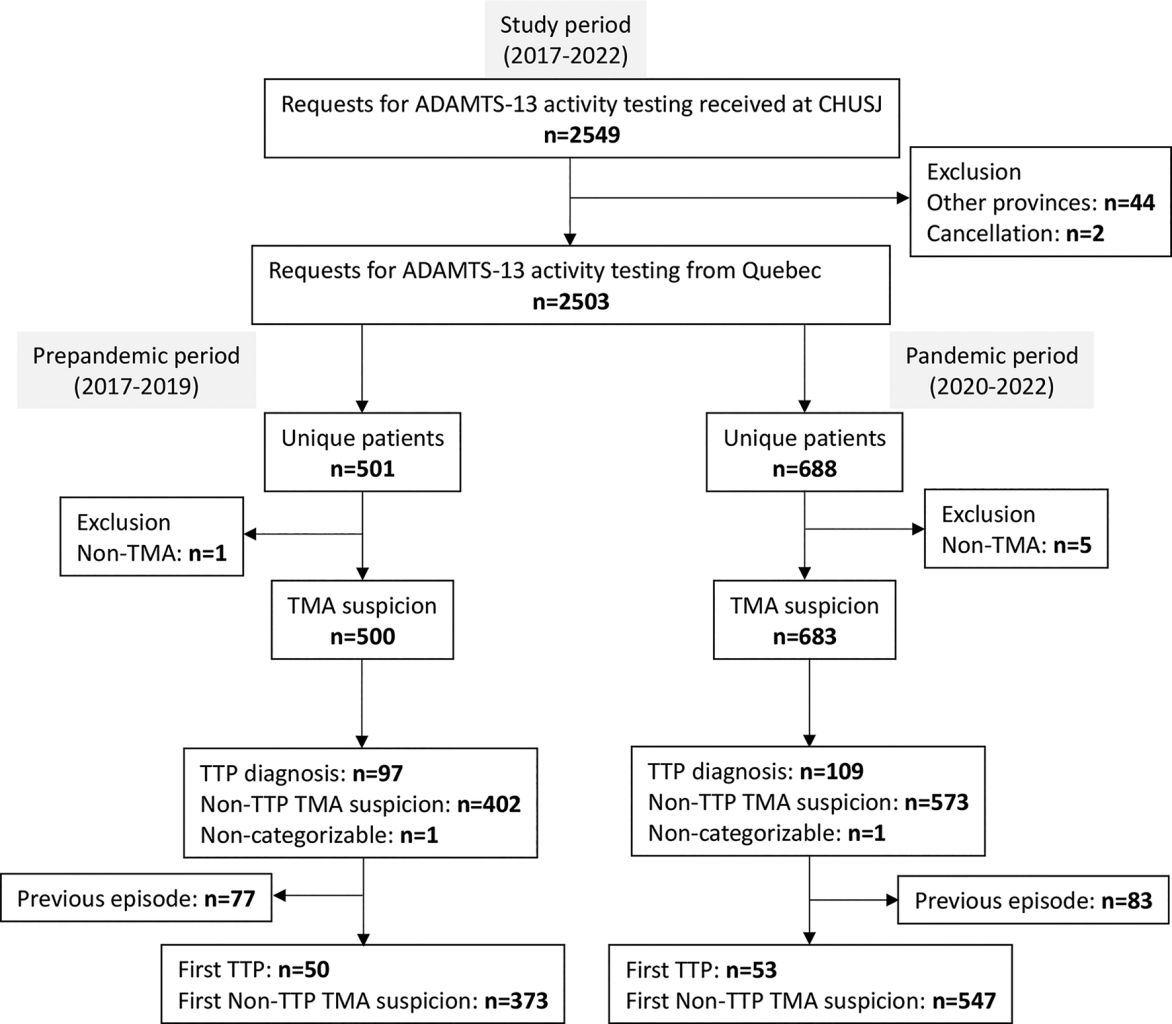
Study design flowchart and patient categorization. ADAMTS-13 a disintegrin and metalloprotease with thrombospondin type 1 repeats, member 13, TMA thrombotic microangiopathy, TTP thrombotic thrombocytopenic purpura. Non-categorizable: patients with a suspected TMA and ADAMTS13 activity > 10% for whom the timing between plasmapheresis and plasma sample collection was unknown

A total of 2549 requisitions for ADAMTS-13 activity testing were received at CHUSJ during the study period (2017–2022) (Fig. 1). A total of 501 and 688 unique patients were identified during the pre-pandemic and pandemic periods, respectively. Five patients were excluded due to referral for a family history of TTP (1 during the pre-pandemic period and 4 during the pandemic). Additionally, one patient was excluded during the pandemic due to a suspicion of disseminated intravascular coagulation (DIC) rather than TMA. In total, 50 patients with supported TTP and 373 with suspected non-TTP TMA were included in the incidence calculation for the pre-pandemic period. For the pandemic period, 53 patients with supported TTP and 547 patients with suspected non-TTP TMA were included in the incidence calculation.

### Temporal trends in the number of ADAMTS-13 activity tests requested and patients referred for ADAMTS-13 testing

The number of ADAMTS-13 tests performed and of patients referred for acute diagnosis, follow-up or longitudinal monitoring are presented in supplementary Fig. 1. Test volume rose by 22% in 2019 (*n* = 395), and by 19% in both 2020 (*n* = 471) and 2022 (*n* = 551) compared to the respective previous years, while remaining stable in 2021 (*n* = 462) compared to 2020. The total number of patients referred for ADAMTS-13 activity measurement increased from 160 in 2017 to 269 in 2022. Although referrals remained steady in 2020, TTP patients accounted for 29% of those tested for ADAMTS-13 activity that year (*n* = 64/219), compared to 24% in 2019 (*n* = 53/217). The normalized test rate for TTP patients was 3.7 in 2017, 3.6 in 2018, 4.0 in 2019, 4.7 in 2020, 4.1 in 2021 and 6.5 tests per patient in 2022.

### Clinical and laboratory features of patients at first presentation

The demographic, clinical, and laboratory characteristics of individuals included in this study are presented in Table 1. The number of patients for whom clinical data were available varied for each parameter, depending on the completeness of medical questionnaires filled out by the requesting physicians. 79% of questionnaires were available during the pre-pandemic period, increasing to 89% during the pandemic for TTP patients. For suspected non-TTP TMA patients, 65% of questionnaires were available in both periods. There were no significant differences in clinical presentation or laboratory results of TTP and suspected non-TTP TMA patients between the two periods. Most TTP cases were observed in female patients with a significantly lower median age during the pandemic compared to the pre-pandemic period (41.5 years; IQR = 31.5–54 vs. 51.5 years; IQR = 42-68.25 respectively; *p* = 0.032; δ = 0.312). The most common symptom at presentation for TTP patients was neurological manifestations, observed in 41% of patients during the pre-pandemic period and 61% during the pandemic (p = 0.059). In contrast to TTP, the median age of suspected non-TTP TMA patients remained stable during the COVID-19 period compared to the previous period in females (54 years vs. 55 years respectively; p = 0.778) and in males (57 vs. 59 years respectively; p = 0.477).

**Table 1:** Baseline characteristics of patients with newly TMA at presentation during the pre-pandemic and pandemic period

		PRE-COVID-19 period TTP	COVID-19 period TTP	*p*-value	PRE-COVID-19 period Non-TTP TMA	COVID-19 period Non-TTP TMA	*p*-value
**Demographic**							
**Patients (n)**		50	53		373	547	
**Age (years)**							
Mean ± SD		52.6 ± 18.2	48.8 ± 20.9	0.333	51.1 ± 24.4	50.8 ± 23.6	0.579
Median (IQR)		54 (42–68)	47 (34-66.5)		57 (32–70)	56 (33–69)	
** Sex**							
Female, n (%)		30 (60%)	34 (64%)		213 (57%)	284 (52%)	
Male, n (%)		20 (40%)	19 (36%)	0.664	160 (43%)	263 (48%)	0.121
**Clinical presentation**							
Fever		9/34 (26%)	10/40 (25%)	0.885	71/210 (34%)	104/336 (31%)	0.486
Neurological symptoms		16/39 (41%)	30/49 (61%)	0.059	81/216 (37%)	109/347 (31%)	0.137
Abdominal signs		14/37 (38%)	13/43 (30%)	0.473	58/213 (27%)	89/335 (27%)	0.864
**Known etiologies **							
Pregnancy		0/47 (0%)	2/51 (4%)	0.496	15/259 (6%)	24/376 (6%)	0.826
Cancer		5/40 (12%)	5/49 (10%)	0.749	49/260 (19%)	84/377 (22%)	0.294
Infection		7/40 (17.5%)	7/49 (14%)	0.679	49/259 (19%)	67/376 (18%)	0.724
Transplantation		3/40 (7%)	2/50 (4%)	0.652	26/260 (10%)	35/376 (9%)	0.771
Drug-associated		4/40 (10%)	6/49 (12%)	1.000	44/259 (17%)	53/377 (14%)	0.313
**Biologic characteristics **							
Haemolytic anemia		36/39 (92%)	47/47 (100%)	0.089	169/228 (74%)	269/346 (78%)	0.318
Thrombocytopenia		37/39 (95%)	46/47 (98%)	0.588	205/243 (84%)	308/362 (85%)	0.809
**ADAMTS-13 activity**							
Mean ± SD		2.5 ± 4.5	1.3 ± 3.6	0.109	71 ± 49	70 ± 37	0.841
Median (IQR)		0 (0-2.25)	0 (0-0.5)		60 (40–95)	62 (42–90)	

Non-TTP TMA: thrombotic microangiopathy other than TTP (suspected); TMA: thrombotic microangiopathy; TTP: thrombotic thrombocytopenic purpura (supported); SD: standard deviation; IQR: interquartile range

The number of patients for whom clinical data were available is indicated for each parameter

### Incidence of thrombotic microangiopathies before and during the pandemic

Over the entire study period (2017–2022), the mean annual incidence was 2.01 per million persons (95%CI: 1.51–2.53) for TTP and 17.95 per million persons (95%CI: 13.04–22.87) for suspected non-TTP TMA (Table 2). In the latter group, the mean annual incidence in males significantly increased from 12.7 per million persons (95%CI: 6.1–19.3) during the pre-pandemic period to 20.3 per million persons (95%CI: 14.1–26.6) during the pandemic period (*p* = 0.023) (Table 2). In contrast, the mean annual incidence of TTP remained stable between both periods in males (*p* = 0.778) and in females (*p* = 0.691).

**Table 2:** Mean annual incidence per million persons-years

	Pre-COVID-19 period	COVID-19 period	*p*-value
*Suspected Non-TTP TMA *			
Total	14.77 ± 2.7	21.12 ± 4.1	0.101
Female	16.83 ± 3.6	21.93 ± 6.2	0.303
Male	12.71 ± 2.7	20.31 ± 2.5	0.023
*Supported TTP *			
Total	1.99 ± 0.7	2.05 ± 0.3	0.903
Female	2.37 ± 0.8	2.63 ± 0.5	0.691
Male	1.59 ± 0.6	1.47 ± 0.4	0.778

Incidence: number of new cases per million population per year. TTP: thrombotic thrombocytopenic purpura; TMA: thrombotic microangiopathy

**Fig. 2:**
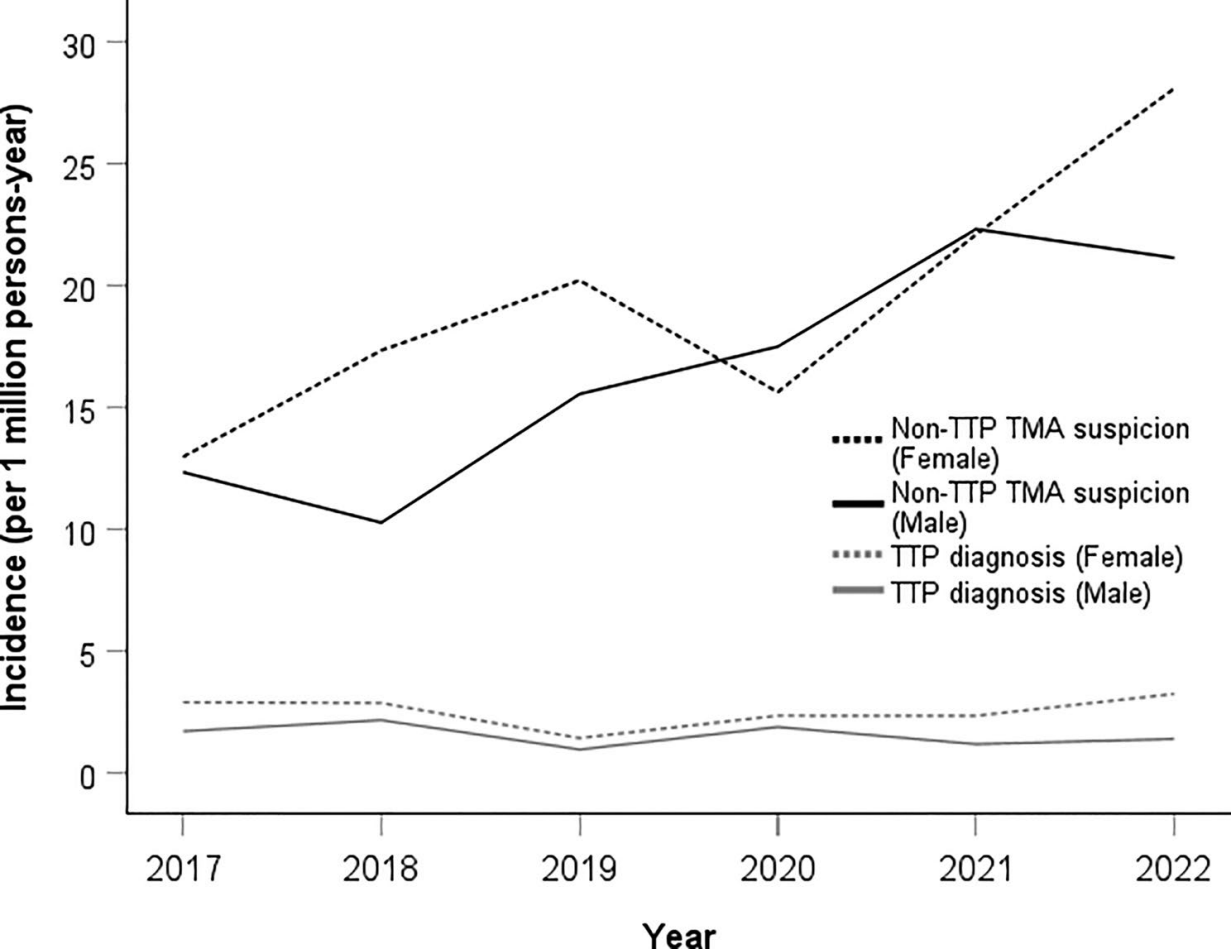
Timeline analysis of the incidence of thrombotic microangiopathy by gender before and during the COVID-19 pandemic

Examining trends over the years, the highest incidence was observed in 2022 (3.22 cases per million persons per year) for female TTP patients and in 2018 (2.15 cases per million persons per year) for male TTP patients (Fig. 2). The peak incidence for suspected non-TTP TMAs occurred in 2022 for females (28.09 cases per million persons per year) and in 2021 for males (22.21 cases per million persons per year).

Subgroup analyses by gender and age (less than 1 to greater than 80 years) (Supplementary Fig. 2) showed a peak in new TTP cases among females aged 30–39 during the pandemic, with a mean annual incidence of 5.99 per million persons (95%CI: 3.5–8.5) compared to 1.21 per million persons (95%CI: -1.4-3.8) pre-pandemic. After adjusting for multiple comparisons, this difference was not statistically significant (*p* = 0.333). The number of suspected non-TTP TMA patients was higher across most age groups for both females and males during the COVID-19 period, especially in males aged 50–59 years (*n* = 50 vs. *n* = 22). The mean annual incidence increased from 11.9 per million persons (95%CI: 10.0-13.7) to 28.7 per million persons (95%CI: 15.9–41.2) during the pandemic in this sub-group (*p* = 0.05).

### Temporal relationship between newly COVID-19-related hospitalizations and thrombotic microangiopathy cases

**Fig. 3:**
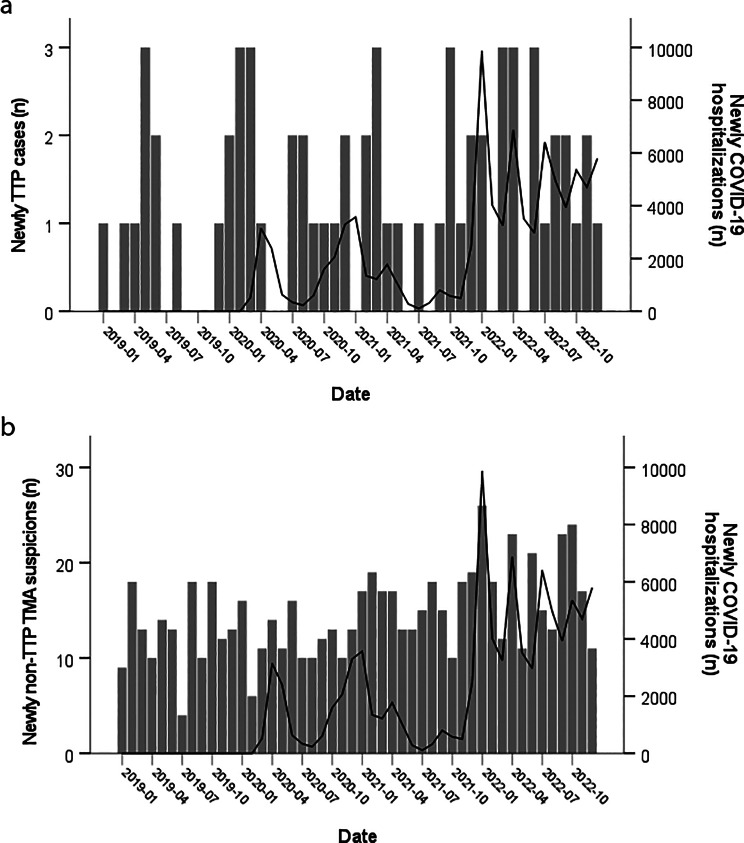
Monthly COVID-19 hospitalizations and number of new thrombotic microangiopathy from 2019 to 2022 in Quebec. **a** Thrombotic thrombocytopenic purpura (TTP) diagnosis; **b** Thrombotic microangiopathy other than TTP (non-TTP TMA) suspicion. The bars represent the number of new TTP cases or non-TTP TMA suspicions. The line indicates the number of new COVID-19 hospitalizations

TMA is a medical emergency that typically requires intensive hospital treatment. Unlike TTP (Fig. 3a), the number of suspected non-TTP TMA cases increased in number at the time of increased new hospitalizations with COVID-19 infection (January 2022) (Fig. 3b). Cross-correlation analyses were then performed to determine whether a temporal relationship exists between the number of new COVID-19-related hospitalizations and newly diagnosed TTP or suspected non-TTP TMA (Supplementary Fig. 3). A weak correlation was found at a lag of 10 weeks (*r* = 0.238) for new TTP cases and at a lag of 1 week for new non-TTP TMA suspicions (*r* = 0.258).

### Profile of patients with COVID-19-associated thrombotic microangiopathy at presentation

Complete or partial clinical data were available for 443 patients of 683 referred for TMA suspicions during the pandemic. Among these 443 patients, 17 cases of SARS-CoV-2 infection were reported as an associated condition at presentation (Table 3). The median age was 53 years (range: 38 to 79 years; IQR = 42–69). Males were over-represented, with a female-to-male ratio of 1:3.25. No patients, except one male aged 50 years, had previously reported TMA episodes. ADAMTS-13 measurement was not available for one patient who died before the test could be performed. Three patients had an ADAMTS-13 activity ≤ 10% and a positive anti-ADAMTS-13 immunoglobulin G (IgG) titer. All of these patients exhibited hemolytic anemia, thrombocytopenia and neurological signs such as confusion and convulsion. Two patients had abdominal pain, and one had fever at presentation. One 38-year-old female had a possible systemic lupus erythematosus (SLE) as an associated condition. The remaining 13 patients had an ADAMTS-13 activity > 10%. Among the 12 patients with available clinical data, 9 had hemolytic anemia, and 10 had thrombocytopenia at presentation. Fever and neurological signs were the most frequently reported clinical features (7/12 and 4/12 cases, respectively), while abdominal signs and kidney damage were observed in 2/12 persons. The majority of patients had no associated conditions other than COVID-19 infection. Two males, aged 50 and 66 years, had cancer, and one female, aged 51 years, had transplant-associated TMA. One 74-year-old male required dialysis. Additionally, a 42-year-old female was suspected to have developed non-TTP TMA 4 months after Sars-CoV-2 infection. She had de novo C3 glomerulonephritis (C3GN) post-kidney transplantation for type I diabetes as an associated condition. Another 30-year-old female received a COVID-19 vaccination prior to being referred for TMA suspicion, with the timing between the vaccination and the onset of TMA symptoms being unknown. At presentation, she had a low ADAMTS-13 activity (4%) and a positive anti-ADAMTS-13 IgG titer. Neither of these two patients had documented previous episodes of TMA.

**Table 3:** Clinical characteristics of patients with TMA associated with COVID-19

Age/sex (y)	Presenting symptoms	Medical history	ADAMTS-13 activity
79/M	Fever Hemolytic anemia/Thrombocytopenia	None	*
38/F	Fever Hemolytic anemia/ Thrombocytopenia Neurological signs (confusion) Abdominal signs (pain)	SLE	0
53/F	Fever Hemolytic anemia/ Thrombocytopenia	None	62
68/M	Fever Thrombocytopenia	None	23
53/M	Thrombocytopenia	None	60
38/M	Unknown	None	84
70/M	Hemolytic anemia/ Thrombocytopenia Neurological signs (convulsions)	None	1
74/M	Fever Hemolytic anemia/ Thrombocytopenia Neurological signs Dialysis	None	70
50/M	Hemolytic anemia/ Thrombocytopenia Neurological signs (confusion) Abdominal signs	Cancer	47
73/M	Fever Hemolytic anemia	None	83
41/M	Hemolytic anemia/Thrombocytopenia Neurological signs	None	78
47/F	Hemolytic anemia/ Thrombocytopenia Neurological signs (confusion) Abdominal signs (pain)	None	0
44/M	Fever Hemolytic anemia/Thrombocytopenia	None	82
66/M	Hemolytic anemia/Thrombocytopenia	Cancer	32
51/F	TMA at renal graft biopsy	Kidney transplantation	59
40/M	Fever Thrombocytopenia	None	30
58/M	Fever Thrombocytopenia Neurological signs (obsession) Abdominal signs (diarrhea)	None	74

SLE: Systemic Lupus Erythematosus; TMA: thrombotic microangiopthy

## Discussion

This retrospective study aimed to determine whether the pandemic impacted the incidence and the clinical presentation of patients with TMA indicators in Quebec.

During the pandemic, the incidence rate of TTP, averaging 2 cases per million people per year, aligned with pre-pandemic trends reported in Canada [36–39]. The median age of female patients was lower during the pandemic compared to the pre-pandemic period. While a causal link with COVID-19 cannot be established, it is noteworthy that a large-scale U.S. study revealed that COVID-19-associated TTP patients tended to be younger than other hospitalized with COVID-19 [43]. Additionally, most reported cases of COVID-19-associated TTP occurred in females [23]. The clinical presentation of TTP patients remained comparable between the two periods, with neurological manifestations being the most frequently recorded symptoms (41% during the pre-pandemic period and 61% during the pandemic). The observed trend toward increased neurological symptoms during the pandemic should be interpreted with caution, as it may reflect improved data capture rather than a true change in clinical presentation. Importantly, these values are within the range reported in pre-pandemic studies (39–80%) [44]. Symptoms such as headache, confusion, and seizures are common in TTP (occurring in roughly 44% of cases) [45] and have also been associated with COVID-19 [46, 47].

Unlike TTP, the incidence of suspected non-TTP TMA appeared to increase during the pandemic, especially among males, rising from 12.7 to 20.3 cases per million people per year, particularly affecting those aged 50–59. Over an eight-year period from 2012 to 2019, the incidence of suspected non-TTP TMA in Quebec was found to be 10.2 cases per million per year [37]. Although a direct causal link between TMA and COVID-19 is challenging to confirm, microvascular thrombi were observed in multiple organs upon autopsy of COVID-19 patients early in the pandemic [19]. Skin and lung tissue examinations from 5 patients with severe COVID-19 revealed deposition of C5b-9, C4d, and mannose-binding protein-associated serine protease 2 (MASP-2) in the microvasculature, consistent with complement-mediated microvascular injury [48]. From the onset of the pandemic to February 2022, 46 COVID-19-associated TMA cases were reported, including 28 complement-mediated cases [49]. Interestingly, suspected non-TTP TMA cases peaked alongside COVID-19 hospitalizations in January 2022. We employed cross-correlation analysis as an exploratory tool to identify potential temporal relationships between COVID-19 hospitalizations and new cases of TMA. The analysis suggested a lagged relationship between COVID-19 hospitalizations and new TMA referrals, but these findings should be interpreted with caution due to the weak correlation coefficients and the potential influence of unidentified confounding factors. Nevertheless, the one-week lag for non-TTP TMA is consistent with reports of COVID-19-associated TMA occurring from the onset of infection up to a month post-viral clearance [49]. This suggests a possible temporal relationship, but it is not sufficient to establish causality between these two events. Regarding TTP, the 10-week lag suggests COVID-19 hospitalizations may not be a direct factor in new TTP cases in our cohort. Indeed, based on case reports, there is a mean of 10 days from COVID-19 symptoms to TTP diagnosis [23]. However, we cannot rule out that COVID-19 could predispose individuals to TTP through mechanisms such as ADAMTS-13 antibody generation [49].

In our study, 17 patients referred for TMA suspicion had COVID-19 as an associated condition. Based on ADAMTS-13 activity measurements, most were classified as having TMA other than TTP. None of these Sars-CoV-2 infected patients were identified with a concurrent bacterial infection, suggesting a likely diagnosis of CM-TMA rather than HUS. However, further investigations, including testing for Shigatoxin-producing *Escherichia coli*, complement gene mutations, and anti-complement factor antibodies [3, 50] would be necessary to differentiate HUS from CM-TMA. Notably, we observed no major differences in the clinical presentation of suspected non-TTP TMA cases associated with COVID-19 compared to pre-pandemic cases. This aligns with a review reporting no substantial differences in the clinical presentation of 46 adults with COVID-19-associated TMA [49]. In contrast, a systematic review of 11 patients with COVID-19-associated TTP highlighted an atypical presentation, with none reporting fever and only 27.3% presenting with neurological symptoms [23]. Due to the limited number of TTP patients with COVID-19 in our study, we were unable to draw conclusions regarding their clinical presentation.

The strength of this study is that we analyzed data for an entire province of Canada. However, our study has limitations. Unlike TTP, non-TTP TMA incidence deduced from our laboratory data is an estimate based on physician suspicions, lacking confirmatory diagnoses. Clinical features, such as platelet count, presence of schistocytes and markers of complement activation, would have helped to distinguish true TMA from TMA-like presentations. Moreover, the publication of COVID-19-associated TMA case reports and studies suggesting a possible role of ADAMTS-13 in the pathogenesis of SARS-COV-2 [51, 52] may have raised awareness, potentially increasing referrals for ADAMTS-13 activity testing. Anemia and thrombocytopenia, hallmarks features of TMA, were also frequently reported in COVID-19 patients [53]. Although a retrospective registry-based study of 100 hospitalized patients with COVID-19 found that only 9% of those with thrombocytopenia met the criteria of TMA [54], this may still have contributed to increase referral for TMA suspicions. Additionally, men around 60 years old were particularly affected by COVID-19 early in the pandemic. In Montreal, the epicenter of the pandemic in Canada, critically ill COVID-19 patients admitted to intensive care units had a median age of 62, with 67% being male [55], potentially increasing referrals for ADAMTS-13 activity testing among males in this age group in Quebec. Conversely, public health restrictions implemented during the first two years of the pandemic to limit Sars-CoV-2 transmission may have reduced infections, some triggering TMA [9]. Finally, it is unclear whether all COVID-19 infections were consistently reported in the medical questionnaires. This may have led to an underestimation of COVID-19-associated TMA cases in our study.

In conclusion, the overall incidence of TTP did not change during the COVID-19 pandemic. The incidence of suspected non-TTP TMA rose among men. Further studies are needed to clarify the relationship between TMA and COVID-19 in specific population groups and to differentiate true TMA from TMA-like presentations to refine incidence calculations.

## Electronic Supplementary Material

Below is the link to the electronic supplementary material.

Supplementary Material 1

## Data Availability

The authors declare that the key data supporting the findings of this study are available within the article. The individual patient data cannot be shared openly to protect study participant privacy. Further data are available from the corresponding author upon reasonable request.

## Abbreviations

ADAMTS-13: A disintegrin and metalloproteinase with a thrombospondin type 1 motif, member 13
aHUS: Atypical hemolytic uremic syndrome
CCF: Cross-correlation function
CHUSJ: CHU Sainte-Justine
CM-TMA: Complement mediated-thrombotic microangiopathy
COVID-19: Coronavirus disease-2019
DIC: Disseminated intravascular coagulation
HUS: Hemolytic uremic syndrome
INESS: Institut national d’excellence en santé et services sociaux
INSPQ: Institut national de santé publique du Québec
IQR: Interquartile range
MAHA: Microangiopathic hemolytic anemia
MASP-2: Mannose-binding protein-associated serine protease 2
PPP: Platelet-poor-plasma
SARS-COV-2: Severe acute respiratory syndrome coronavirus 2
SLE: Systemic Lupus Erythematosus
STEC-HUS: Shiga toxin-producing Escherichia hemolytic uremic syndrome
TMA: Thrombotic microangiopathy
TPE: Therapeutic plasma exchange
TTP: Thrombotic thrombocytopenic purpura
VEGF: Vascular endothelial growth factor
VWF: Von Willebrand factor
