# Supplementary figures and images for: The impact of the COVID-19 pandemic on incidence and clinical presentation of thrombotic microangiopathies: data from a laboratory centralizing ADAMTS-13 testing in Quebec
Source: Orphanet J Rare Dis. 2025 Sep 24;20:480. doi: 10.1186/s13023-025-03960-5 (PMC12462063; doi:10.1186/s13023-025-03960-5)

Supplementary Figure 1


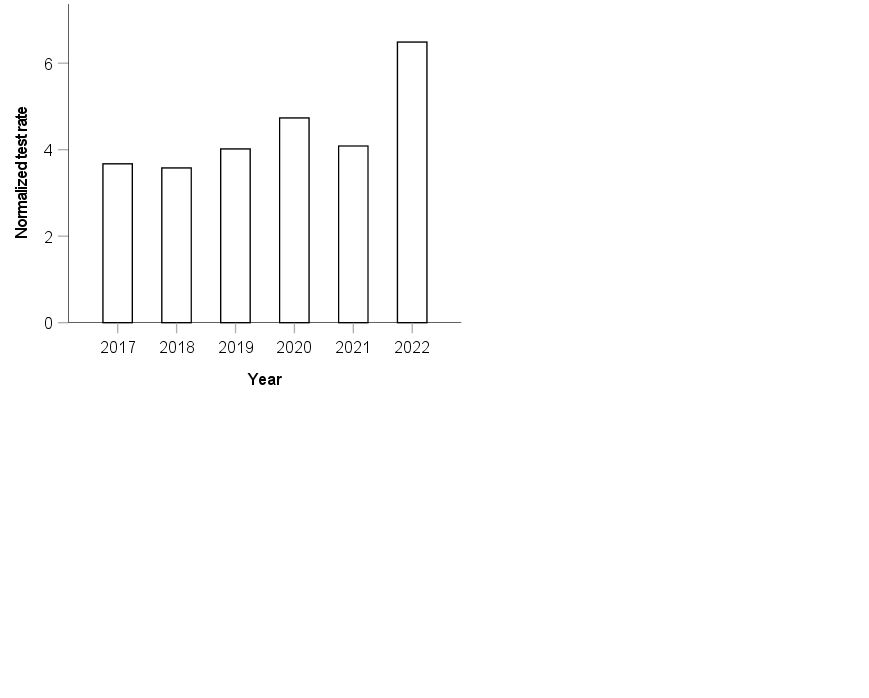

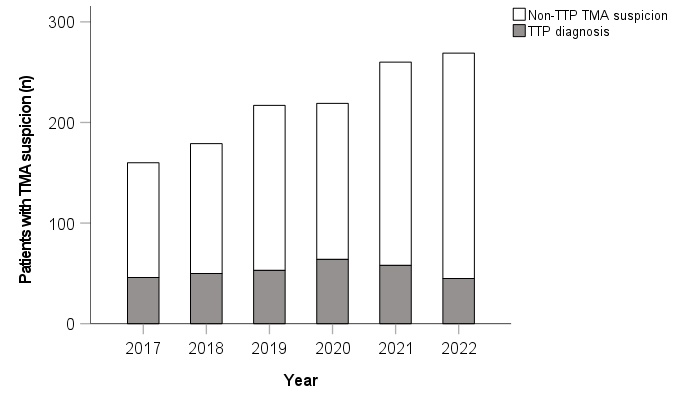

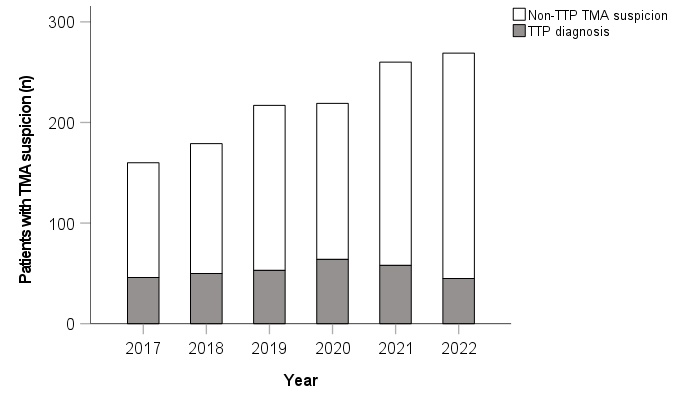

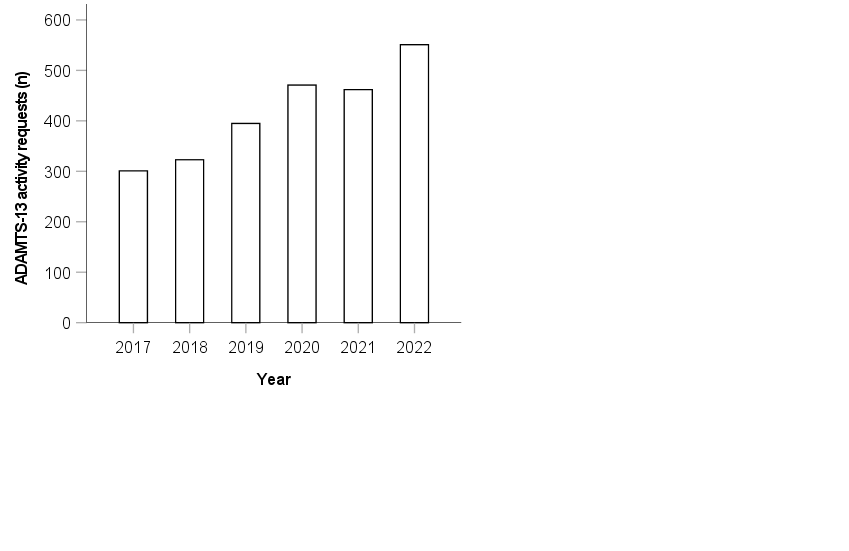


b

c

a

Supplement: Supplementary file 1 — Supplementary Material 1 [file 13023_2025_3960_MOESM1_ESM.zip › 13023_2025_3960_MOESM1_ESM/Supplementary Figure 1.docx]

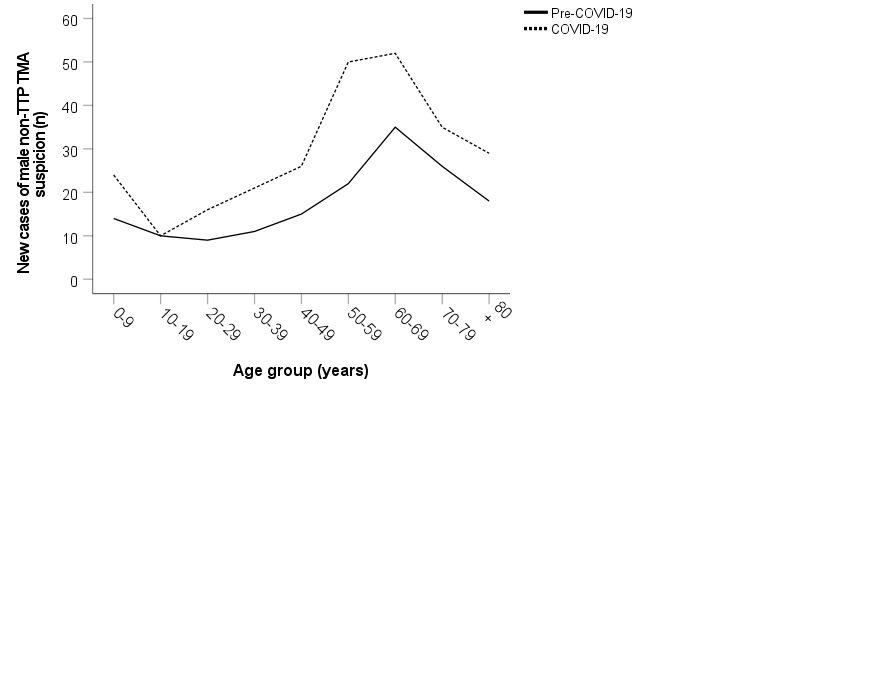

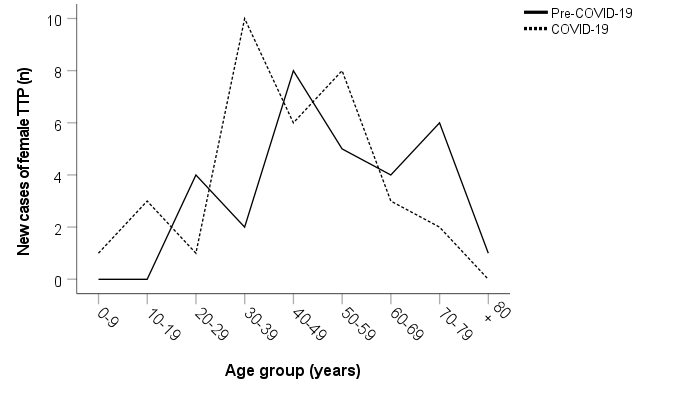
Supplementary Figure 2

b

d

c

a


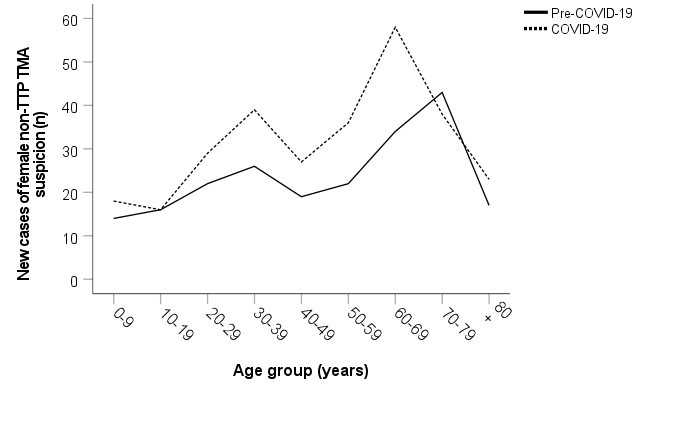

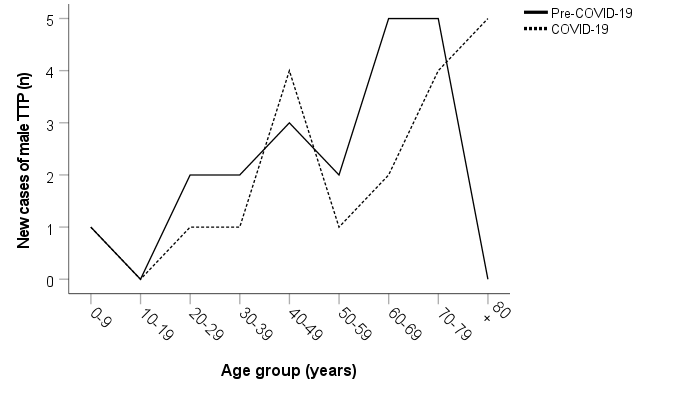

Supplement: Supplementary file 1 — Supplementary Material 1 [file 13023_2025_3960_MOESM1_ESM.zip › 13023_2025_3960_MOESM1_ESM/Supplementary Figure 2.docx]

**
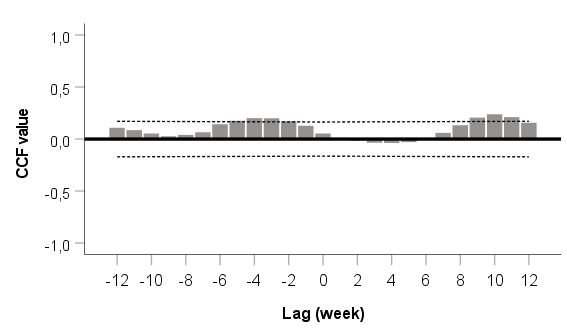
**Supplementary Figure 3

a

**
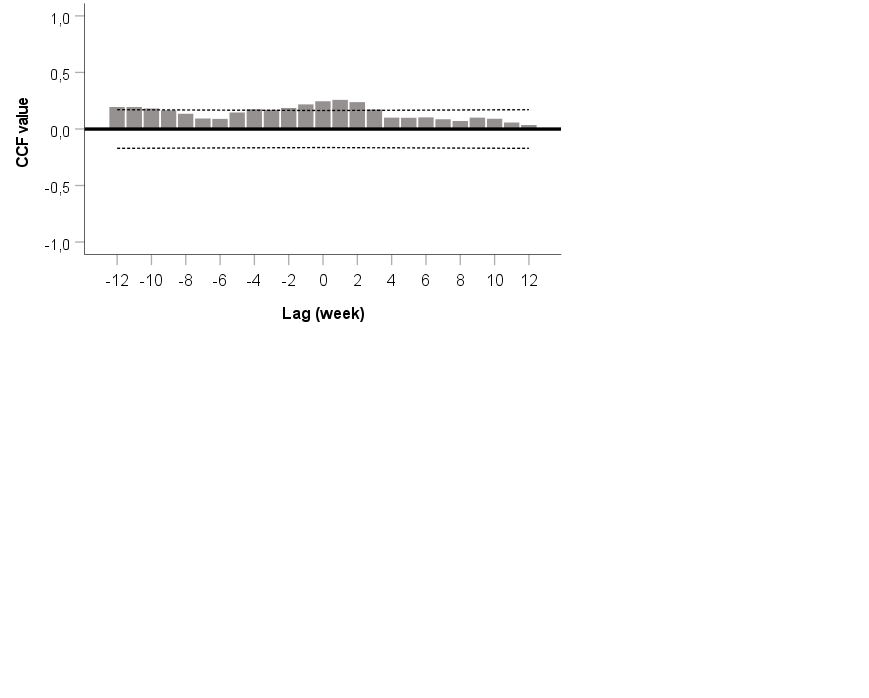
**

b

Supplement: Supplementary file 1 — Supplementary Material 1 [file 13023_2025_3960_MOESM1_ESM.zip › 13023_2025_3960_MOESM1_ESM/Supplementary Figure 3.docx]
